# Universal Multiplex PCR: a novel method of simultaneous amplification of multiple DNA fragments

**DOI:** 10.1186/1746-4811-8-32

**Published:** 2012-08-15

**Authors:** Daxing Wen, Chunqing Zhang

**Affiliations:** 1 State Key Laboratory of Crop Biology, Agronomy College, Shandong Agricultural University, Tai’an, Shandong Province 271018, P. R. China

**Keywords:** Multiplex PCR, Polymorphisms, Species, Genetic purity, Maize

## Abstract

**Background:**

Multiplex PCR has been successfully applied in many areas since it was first reported in 1988; however, it suffers from poor universality.

**Results:**

A novel method called Universal Multiplex PCR (UM-PCR) was created, which simultaneously amplifies multiple target fragments from genomic DNA. The method has two steps. First, the universal adapter-F and universal adapter-R are connected to the forward primers and the reverse primers, respectively. Hairpin structures and cross dimers of five pairs of adapter-primers are detected. Second, UM-PCR amplification is implemented using a novel PCR procedure termed “Two Rounds Mode” (three and 28–32 cycles). The first round (the first three cycles) is named the “One by One Annealing Round”. The second round (28–32 cycles) combines annealing with extension. In the first two cycles of the first round, primers only amplify the specific templates; there are no templates for the universal adapters. The templates of universal adapters begin to be synthesized from the second cycle of the first round, and universal adapters and primers commence full amplification from the third cycle of the first round.

**Conclusions:**

UM-PCR greatly improves the universality of multiplex PCR. UM-PCR could rapidly detect the genetic purity of maize seeds. In addition, it could be applied in other areas, such as analysis of polymorphisms, quantitative assays and identifications of species.

## Background

Maize, as one of the three major grain crops worldwide, is important for feed and industrial raw materials. It occupies an important position in world agricultural production. The genetic purity of maize seeds has received much attention. Low seed purity reduces the biomass, thereby affecting the final yield.

There are many methods for testing the genetic purity of seeds, such as morphological identification, identification by physical and chemical methods, identification by physiological and biochemical methods, and identification by molecular biology and cell biology [1-15]. Currently, protein and isozyme electrophoresis are widely used in seed genetic purity testing. Traditional detection techniques still have an important role; however, most of them are time and labor intensive, and are vulnerable to environmental conditions. Thus, they struggle to meet the requirements of rapid and accurate testing of seed genetic purity. Modern molecular biology and information technology have been applied to the testing of seed genetic purity, with the aim of establishing a rapid and accurate indoor test method. Current research has focused on machine vision (i.e. imaging-based automatic inspection) [1,16-23] and simple sequence repeat (SSR) detection [4,5,8,11,14,24].

Over the last decades, the use of molecular techniques to detect seed genetic purity has been increasingly advocated. Typically, polymerase chain reaction (PCR) is applied to amplify short fragments of DNA from samples, and the PCR products are then identified by gel electrophoresis. The main PCR-based methods for testing seed genetic purity are random amplified polymorphic DNA (RAPD), restriction fragment length polymorphism (RFLP), amplified fragment length polymorphism (AFLP) and detection of SSRs [2,4,5,14]. However, these methods may have one or more disadvantages, including inferior reproducibility, low efficiency or high cost.

Multiplex PCR [25-46] has been successfully applied in many areas (e.g., analyses of mutations and polymorphisms, quantitative assays and identifications of species) since it was first reported in 1988 [47]. It can simultaneously amplify primer mixtures, thereby decreasing the detection cost and overcoming the weakness of single PCR detection, which only amplifies a pair of primers once [26]. Multiplex PCR requires three to five pairs of SSR primers for testing maize seed genetic purity. Multiplex PCR can complete the test in a single reaction. However, multiplex PCR has several disadvantages, such as complexity, low amplification efficiency, variable efficiency on different templates and poor universality, which restrict its commercial application [44].

The aim of this study was to develop a rapid and accurate molecular identification method for testing the genetic purity of maize seeds. We present a method, termed Universal Multiplex PCR (UM-PCR), which can simultaneously detect five SSRs. UM-PCR greatly improves the universality of multiplex PCR.

## Results

### Validation of the utility of the adapters

**Figure 1:**
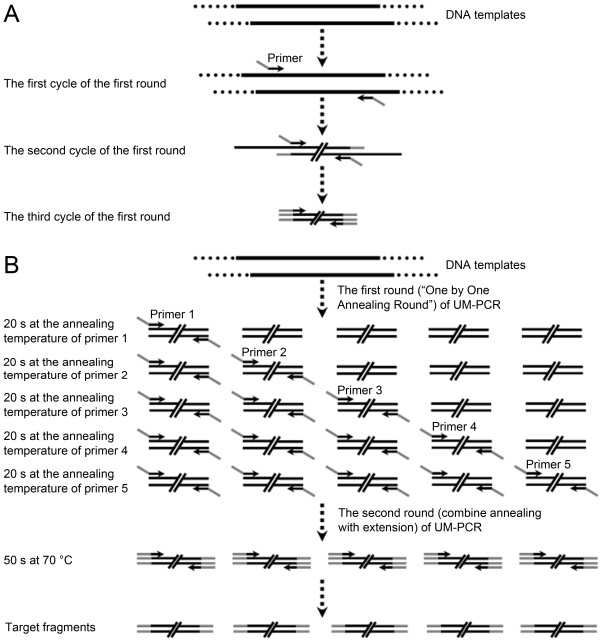
**Schematic representation of adapter-primers and UM-PCR.** (**A**) Schematic representation of adapter-primers. (**B**) Schematic representation of UM-PCR.

**Figure 2:**
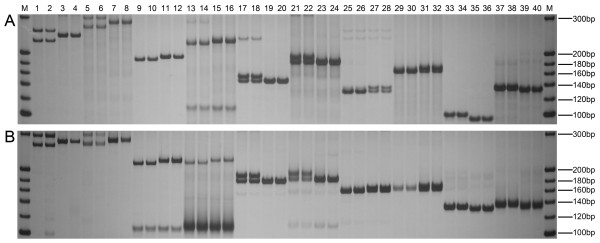
**Comparison of PCR products amplified using SSR primers and different types of adapter-primers.** (**A**) PCR products amplified using SSR primers and U-primers. M, 20 bp DNA Marker (Takara, Dalian, China); lanes 1–4, phi085; lanes 5–8, U-phi085; lanes 9–12, phi041; lanes 13–16, U-phi041; lanes 17–20, phi123; lanes 21–24, U-phi123; lanes 25–28, umc1478; lanes 29–32, U-umc1478; lanes 33–36, umc1268; lanes 37–40, U-umc1268; Zhengdan 958: lanes 1, 2, 5, 6, 9, 10, 13, 14, 17, 18, 21, 22, 25, 26, 29, 30, 33, 34, 37, 38; Xianyu 335: lanes 3, 4, 7, 8, 11, 12, 15, 16, 19, 20, 23, 24, 27, 28, 31, 32, 35, 36, 39, 40. (**B**) PCR products amplified using U-primers and C-primers. M, 20 bp DNA Marker (Takara); lanes 1–4, U-phi085; lanes 5–8, C-phi085; lanes 9–12, U-phi041; lanes 13–16, C-phi041; lanes 17–20, U-phi123; lanes 21–24, C-phi123; lanes 25–28, U-umc1478; lanes 29–32, C-umc1478; lanes 33–36, U-umc1268; lanes 37–40, C-umc1268; Zhengdan 958: lanes 1, 2, 5, 6, 9, 10, 13, 14, 17, 18, 21, 22, 25, 26, 29, 30, 33, 34, 37, 38; Xianyu 335: lanes 3, 4, 7, 8, 11, 12, 15, 16, 19, 20, 23, 24, 27, 28, 31, 32, 35, 36, 39, 40.

After a series of optimization assays, UM-PCR amplification was implemented using “Two Rounds Mode”. The specific process is described in the methods and shown in Figure 1. Specific fragments representing SSRs were amplified using traditional SSR PCR primers and UM-PCR primers (identified with a U in the following list: phi085, U-phi085, phi041, U-phi041, phi123, U-phi123, umc1478, U-umc1478, umc1268 and U-umc1268) from maize varieties Zhengdan 958 and Xianyu 335 (Figure 2A). The results suggested that Zhengdan 958 and Xianyu 335 could be identified based on the length of PCR products, with no cross-reaction. The bands amplified by “Two Rounds Mode” with U-primers were slightly larger (36 bp) than the bands amplified by routine PCR with SSR primers. The results showed that the amplification efficiency of U-primers was similar to that of SSR primers.

The same SSR fragments were also amplified using common primers as well as universal primers (identified with a C in the following list: U-phi085, C-phi085, U-phi041, C-phi041, U-phi123, C-phi123, U-umc1478, C-umc1478, U-umc1268 and C-umc1268) from Zhengdan 958 and Xianyu 335 (Figure 2B). The bands amplified by “Two Rounds Mode” with C-primers were indistinct and weaker than the bands amplified by “Two Rounds Mode” with U-primers. There were some severe non-specific amplification products in lanes 13–16 amplified by C-phi041, and the target fragments of 13–16 were very weak as a result. There were some slight non-specific amplification products in lanes 9–12 amplified by U-phi041. The results showed that the common adapter gave poorer results than the universal adapters.

### Validation of the utility of UM-PCR

**Figure 3:**
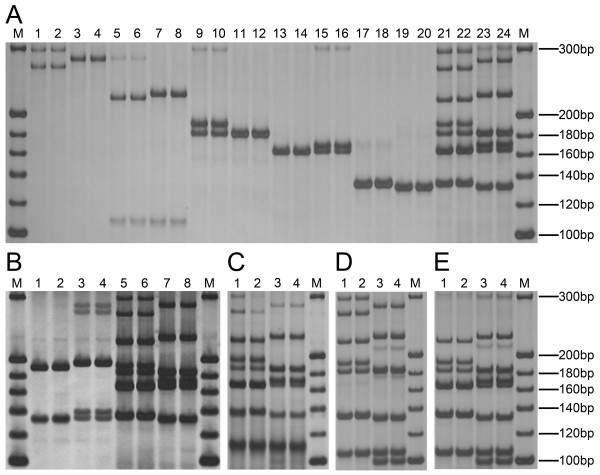
**Comparison of UM-PCR products amplified using SSR primers and different types of adapter-primers.** (**A**) Comparison of single PCR products and quintuple PCR products amplified using U-primers. M, 20 bp DNA Marker (Takara); lanes 1–4, U-phi085; lanes 5–8, U-phi041; from 9 to 12, U-phi123; lanes 13–16, U-umc1478; lanes 17–20, U-umc1268; lanes 21–24 contain five pairs of U-primers (U-phi085, U-phi041, U-phi123, U-umc1478 and U-umc1268). Zhengdan 958: lanes 1, 2, 5, 6, 9, 10, 13, 14, 17, 18, 21, 22; Xianyu 335: lanes 3, 4, 7, 8, 11, 12, 15, 16, 19, 20, 23, 24. (**B**) Comparison of quintuple PCR products amplified using SSR primers and U-primers. M, 20 bp DNA Marker (Takara); lanes 1–4 contain five pairs of SSR primers (phi085, phi041, phi123, umc1478 and umc1268); lanes 5–8 contain five pairs of U-primers (U-phi085, U-phi041, U-phi123, U-umc1478 and U-umc1268); Zhengdan 958: lanes 1, 2, 5, 6; Xianyu 335: lanes 3, 4, 7, 8. (**C**) Quintuple PCR products amplified using C-primers. Lanes 1–4 contain five pairs of C-primers (C-phi085, C-phi041, C-phi123, C-umc1478 and C-umc1268); M, 20 bp DNA Marker (Takara); Zhengdan 958: lanes 1, 2; Xianyu 335: lanes 3, 4. (**D**) Quintuple PCR products amplified using U-primers. Lanes 1–4 contain five pairs of U-primers (U-phi085, U-phi041, U-phi123, U-umc1268 and U-phi120); M, 20 bp DNA Marker (Takara); Zhengdan 958: lanes 1, 2; Xianyu 335: lanes 3, 4. (**E**) Quintuple PCR products amplified using U-primers. Lanes 1–4 contain five pairs of U-primers (U-phi041, U-phi123, U-umc1478, U-umc1268 and U-phi120); M, 20 bp DNA Marker (Takara); Zhengdan 958: lanes 1, 2; Xianyu 335: lanes 3, 4.

Validation of universal quintuple PCR conditions was performed with five pairs of U-primers and compared to the results individual pairs of U-primers. The optimized PCR amplification conditions were the same as those used above. In Figure 3A, amplifications from individual pairs of U-primers are shown in lanes 1–20, while lanes 21–24 show the corresponding quintuple PCR. In lanes 1–2, the bands amplified by U-phi085 were less bright than those amplified in lanes 3–20. The optimized PCR amplification was that the amount of U-phi085 primers should be twice other U-primers. In lanes 21–22, the bands amplified by U-phi085 (the amount of U-phi085 was twice the other U-primers) were almost as bright as those amplified by the other U-primers, but were not as bright as those shown in lanes 1–2. Although the bands of lanes 21–24 were amplified by five pairs of U-primers, they showed more than five bands (SSR is codominant.). The results suggested that UM-PCR could generate clear band patterns using five pairs of U-primers. Accordingly, Zhengdan 958 and Xianyu 335 could be identified based on the length of PCR fragments, with no cross-reaction, by UM-PCR.

The quintuple PCR products that were amplified using five pairs of normal SSR primers (Figure 3B, lanes 1–4) showed bands corresponding to only two SSRs (phi041 and umc1478). The reason may be because the Tms of the two pairs of normal SSR primers is higher than the others, and the annealing temperature of the second round is very high in “Two Rounds Mode”. The quintuple PCR products that were amplified using U-primers were present as clear bands (Figure 3B, lanes 5–8). The quintuple PCR products that were amplified using C-primers (Figure 3C) were not as clear as those amplified using U-primers (Figure 3A, lanes 21–24). The results showed that the C-primers were not optimal for UM-PCR. Similar results are shown in Figure 3D, E, with any differences being caused by the particular U-primer used. Importantly, UM-PCR could be applied for testing the genetic purity of maize seeds.

**Figure 4:**
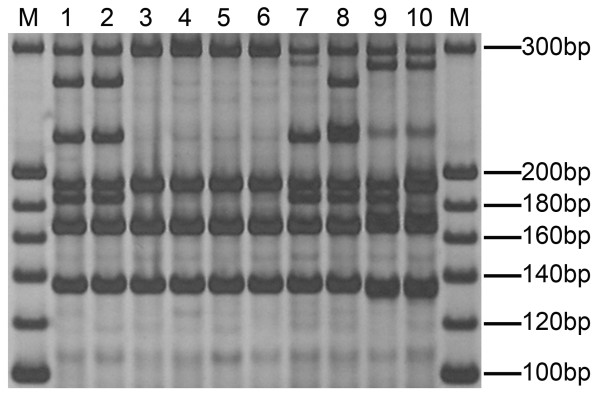
**Gel region showing the detection of genetic purity of a maize seed lot.** M, 20 bp DNA Marker (Takara); lanes 1–10 contain five pairs of U-primers (U-phi085, U-phi041, U-phi123, U-umc1478 and U-umc1268); lanes 1–2, standard bands of Zhengdan 958 (for convenience, the standard bands of only two seeds are shown); lanes 3–6, female parent’s bands of Zhengdan 958; lanes 7–10, the bands generated from off-type seeds.

The genetic purity of a maize seed lot sampled from Zhengdan 958, which is the largest maize cultivar planted in China, was detected by UM-PCR using 200 seeds (Figure 4). Additional file 1 shows this in more detail. Figure 4 shows that 192 seeds were identified as Zhengdan 958, four seeds were identified as the female parent of Zhengdan 958, and four seeds were identified as off-type seeds. In these off-type seeds, U-phi085 showed different band patterns in lanes 7, 9 and 10. U-phi041 showed a different band pattern in lane 8. U-phi123 showed a different band pattern in lane 10. U-umc1478 showed a different band pattern in lanes 9 and 10. U-umc1268 showed a different band pattern in lanes 9 and10. Thus, the genetic purity of the maize seed lot was 96%.

## Discussion

### Adapter-primers design

Novel adapter-primers were designed for UM-PCR in this study. The design of primers is very important for multiplex PCR, because the specificity and melting temperature of the primers are more critical than for routine PCR [26]. Primers with similar annealing temperatures are usually chosen for conventional multiplex PCR. However, for UM-PCR, it is not necessary to consider the differences in annealing temperatures of SSR primers. In this study, two 18 bp adapters are added to the 5’ end of the forward and the reverse SSR primers. The adapters improve the specificity and melting temperature of the SSR primers, and the differences in melting temperatures among the adapter-primers is smaller than for SSR primers. Nevertheless, not all adapters are suitable for UM-PCR, e.g., the common adapter (5’-CCTTCCTTCCTTCCCCCC-3’) is not optimal. This may be because the sequence of the common adapter occurs frequently in the maize genome. The design of adapter-primers needs to consider the sequence of adapters: It is better that the sequence of the adapters is rare. The annealing temperature of adapter-primers reaches about 70°C. In addition, the formation of hairpin structures and cross dimers among the five pairs of primers should be minimized. There should be no more than six consecutive complementary base pairs. UM-PCR could detect more than 10 pairs of SSR primers simultaneously if the adapter-primers are fluorescently labeled.

### The amplification procedure of UM-PCR design

After optimization, UM-PCR amplification was performed in “Two Rounds Mode”. The One by One Annealing Round (the first three cycles) is different from touchdown PCR. In the first two cycles of the first round, primers bind to the specific maize sequences in the templates, whereas universal adapters do not have any target templates. The templates for the universal adapters begin to be synthesized from the second cycle of the first round, and universal adapters and primers commence full amplification of the templates from the third cycle of the first round. The reason why touchdown PCR is not used in UM-PCR is that it would increase non-specific amplification products. Disproportionate amplification cannot be avoided among different primers in conventional multiplex PCR, because the amplification efficiencies of primers may be different in the linear and exponential amplification phases [45]. However, UM-PCR partly solves the problem using the “Two Rounds Mode” and reduces non-specific amplification products.

### The superiority of UM-PCR

First, UM-PCR can partly overcome the disadvantages of conventional multiplex PCR, such as low amplification efficiency and variable efficiency on different templates. Conventional multiplex PCR does not usually result in equal signals from multiple target fragments [26,44]. However, the adapter-primers are designed to amplify DNA templates at a similar efficiency using the “Two Rounds Mode” and by adjusting the amount of adapter-primers. UM-PCR can also overcome the shortcomings of traditional maize seed genetic purity testing methods, such as long processing time; the traditional tests are affected by human factors, and are vulnerable to the impact of environmental conditions [8,9,13]. Using the UM-PCR method, five SSR loci can be identified once, and it is easier and more sensitive than conventional methods. Last, the universality of conventional multiplex PCR is poor, and some technical problems remain unsolved [41]. However, UM-PCR greatly improves the universality of multiplex PCR. The use of universal adapters and the “Two Rounds Mode” means that UM-PCR is not only good for testing the genetic purity of maize seeds, but can also be widely applied in other areas (e.g., analyses of polymorphisms, quantitative assays and identifications of species). Further research to develop a rapid detection technique of the genetic purity of maize seeds that is combined with UM-PCR is ongoing.

## Conclusions

The novel method, UM-PCR, which involves the design of adapter-primers and the establishment of the “Two Rounds Mode”, can be used for the identification five SSR loci simultaneously, and could potentially be used to identify more than five SSR loci. Thus, it can be widely applied in multiplex PCR assays, especially in testing the genetic purity of seeds.

## Methods

### Materials

Maize cultivars Zhengdan 958 and Xianyu 335 were used.

### DNA extraction

DNA was extracted from the maize dry seeds (Zhengdan 958 and Xianyu 335) for seed genetic purity testing. A seed was crushed and then placed in a 1.5 mL centrifuge tube. We then preheated 500 μL DNA extracting solution to 65°C (0.5 M NaCl, 100 mM Tris–HCl pH 8.0, and 50 mM EDTA, 5% SDS, 1% PVP) and added this. The solution was mixed and incubated at 65°C for 45 min. The sample was then centrifuged at 10 000 g for 10 min at room temperature. The supernatant was transferred to a fresh centrifuge tube. An equal volume of phenol/chloroform/isoamyl alcohol (25:24:1 v/v/v) was added and mixed. The sample was centrifuged at 10 000 g for 10 min at room temperature, and the supernatant was transferred to a fresh centrifuge tube. An equal volume of chloroform/isoamyl alcohol (24:1 v/v) was added and mixed. The sample was centrifuged again and the supernatant removed to a fresh tube. An equal volume of isopropanol was added and stood at room temperature for 3 min. The sample was centrifuged at 10 000 g for 5 min and the supernatant was discarded. The pellet was washed twice with 150 μL 70% ethanol and air dried before being resuspended in 50 μL 1 × TE (10 mM Tris–HCl pH 8.0, 0.1 mM EDTA) buffer. The DNA solution was used directly as the template for PCR amplification or stored at −20°C.

### Adapter-primers design

In this study, universal adapters were designed. Universal adapter-F (5’-CTCGTAGACTGCGTACCA-3’) and universal adapter-R (5’-TACTCAGGACTCATCGTC-3’) designed based on the *Eco*RI-adapter and the *Mse*I-adapter of AFLP, respectively [48]; however, their sequences are not identical to the AFLP adapters. Both universal adapters were single stranded. Universal adapter-F and universal adapter-R were linked to the 5’ end of the forward and reverse SSR primers, respectively (U-primers include universal adapters and SSR primers). In this way, the difference of annealing temperature among the primers could be reduced, and the annealing temperature of adapter-primers would reach about 70°C. The designed primers were checked for their propensity to form hairpin structures and cross dimers, which should be minimized. To verify the universality of UM-PCR, we selected some highly specific SSR primers whose annealing temperatures were different. Among these primers, the lowest annealing temperature was about 10°C lower than the highest annealing temperature. To verify the feasibility of the adapters, we also tested a common adapter (5’-CCTTCCTTCCTTCCCCCC-3’) that was taken from reference [26]. The adapters and primers are shown in Table 1.

**Table 1:** Sequences of adapters and primers

**Name**	**Sequence (5' to 3')**	**Tm (°C)**	**References**
universal adapter-F	**CTCGTAGACTGCGTACCA**	57.30	This study
universal adapter-R	**TACTCAGGACTCATCGTC**	55.02	This study
common adapter	**CCTTCCTTCCTTCCCCCC**	61.86	Bai et al., 2009
phi085-F	AGCAGAACGGCAAGGGCTACT	61.92	MaizeGDB
phi085-R	TTTGGCACACCACGACGA	57.30	MaizeGDB
U-phi085-F	**CTCGTAGACTGCGTACCA**AGCAGAACGGCAAGGGCTACT	72.61	This study
U-phi085-R	**TACTCAGGACTCATCGTC**TTTGGCACACCACGACGA	70.05	This study
C-phi085-F	**CCTTCCTTCCTTCCCCCC**AGCAGAACGGCAAGGGCTACT	74.71	This study
C-phi085-R	**CCTTCCTTCCTTCCCCCC**TTTGGCACACCACGACGA	73.47	This study
phi041-F	TTGGCTCCCAGCGCCGCAAA	63.95	MaizeGDB
phi041-R	GATCCAGAGCGATTTGACGGCA	61.94	MaizeGDB
U-phi041-F	**CTCGTAGACTGCGTACCA**TTGGCTCCCAGCGCCGCAAA	73.96	This study
U-phi041-R	**TACTCAGGACTCATCGTC**GATCCAGAGCGATTTGACGGCA	71.33	This study
C-phi041-F	**CCTTCCTTCCTTCCCCCC**TTGGCTCCCAGCGCCGCAAA	76.12	This study
C-phi041-R	**CCTTCCTTCCTTCCCCCC**GATCCAGAGCGATTTGACGGCA	74.40	This study
phi123-F	GGAGACGAGGTGCTACTTCTTCAA	61.97	MaizeGDB
phi123-R	TGTGGCTGAGGCTAGGAATCTC	61.94	MaizeGDB
U-phi123-F	**CTCGTAGACTGCGTACCA**GGAGACGAGGTGCTACTTCTTCAA	71.87	This study
U-phi123-R	**TACTCAGGACTCATCGTC**TGTGGCTGAGGCTAGGAATCTC	71.33	This study
C-phi123-F	**CCTTCCTTCCTTCCCCCC**GGAGACGAGGTGCTACTTCTTCAA	73.82	This study
C-phi123-R	**CCTTCCTTCCTTCCCCCC**TGTGGCTGAGGCTAGGAATCTC	74.40	This study
umc1478-F	GAAGCTTCTCCTCTCGCGTCTC	63.80	MaizeGDB
umc1478-R	CAGTCCCAGACCCTAGCTCAGTC	65.52	MaizeGDB
U-umc1478-F	**CTCGTAGACTGCGTACCA**GAAGCTTCTCCTCTCGCGTCTC	73.38	This study
U-umc1478-R	**TACTCAGGACTCATCGTC**CAGTCCCAGACCCTAGCTCAGTC	73.10	This study
C-umc1478-F	**CCTTCCTTCCTTCCCCCC**GAAGCTTCTCCTCTCGCGTCTC	75.43	This study
C-umc1478-R	**CCTTCCTTCCTTCCCCCC**CAGTCCCAGACCCTAGCTCAGTC	76.10	This study
umc1268-F	ACGAACAACCTAGCACAGTCCTAAA	60.34	MaizeGDB
umc1268-R	CAAGGCGGTTACCAAGTTTACATC	60.26	MaizeGDB
U-umc1268-F	**CTCGTAGACTGCGTACCA**ACGAACAACCTAGCACAGTCCTAAA	70.70	This study
U-umc1268-R	**TACTCAGGACTCATCGTC**CAAGGCGGTTACCAAGTTTACATC	69.92	This study
C-umc1268-F	**CCTTCCTTCCTTCCCCCC**ACGAACAACCTAGCACAGTCCTAAA	71.93	This study
C-umc1268-R	**CCTTCCTTCCTTCCCCCC**CAAGGCGGTTACCAAGTTTACATC	72.85	This study
phi120-F	TGATGTCCCAGCTCTGAACTGAC	61.92	MaizeGDB
phi120-R	GACTCTCACGGCGAGGTATGA	61.95	MaizeGDB
U-phi120-F	**CTCGTAGACTGCGTACCA**TGATGTCCCAGCTCTGAACTGAC	72.10	This study
U-phi120-R	**TACTCAGGACTCATCGTC**GACTCTCACGGCGAGGTATGA	71.56	This study

Tms are taken from the primer synthesis report card.

### PCR procedure

To verify the reliability of UM-PCR, a series of PCR assays were carried out. All the UM-PCR reactions were conducted using a Biometra PCR system (Biometra, Gottingen, Germany) in a 20 μL reaction volume, which contained 10 μL 2 × Power Taq PCR MasterMix (BioTeke, Beijing, China), 3 μL DEPC H_2_O, 0.5 μL of 10 μM (a total of five pairs of adapter-primers) adapter-primers (Sangon, Shanghai, China) and 2 μL DNA. UM-PCR amplification was implemented using a novel PCR procedure, termed the “Two Rounds Mode” (3 and 28–32 cycles). The first round (the first three cycles) was termed the “One by One Annealing Round”. The second round (28–32 cycles, usually 30 cycles) combines annealing with extension. The final thermal cycling program is shown in Table 2, and the basic principle of the adapter-primers and UM-PCR method is outlined in Figure 1. The routine PCR reactions were also conducted using a Biometra PCR system (Biometra) in a 20 μL reaction volume, which contained 10 μL 2 × Power Taq PCR MasterMix (BioTeke), 7 μL DEPC H_2_O, 0.5 μL of 10 μM primers (Sangon) and 2 μL DNA. The final thermal cycling included an initial 5 min denaturation at 94°C; 30 cycles for 40 s at 94°C, 30 s at the annealing temperature of the primer, and 30 s at 72°C; and a final extension for 10 min at 72°C.

**Table 2:** The UM-PCR procedure (“Two Rounds Mode”)

	**Temperature (°C)**	**Time**
	94	5 min
	94	40s
	annealing temperature of primer 1	20s
	annealing temperature of primer 2	20s
	annealing temperature of primer 3	20s
	annealing temperature of primer 4	20s
	annealing temperature of primer 5	20s
	72	30s
	94	40s
	70	50s
	72	10 min

In the first round (the first three cycles), annealing temperatures that are optimized in accordance with the Tm of normal SSR primers are sorted in descending order. Annealing temperatures may be different in various combinations of U-primers.

### Gel electrophoresis

PCR products were electrophoresed in 9% polyacrylamide gel for 3 h (10 V/cm) in a Tris-Boric acid-EDTA buffer [0.08 M Tri-HCl (pH 8.5), 0.08 M Boric acid and 2.4 mM EDTA]. After electrophoresis, the gel was stained by 0.2% silver and developed by 3% NaOH and 5 mL 37% formaldehyde per liter. The gel was then photographed with a camera.

## Abbreviations

UM-PCR: Universal Multiplex PCR; U-primers: Universal adapter primers contain universal adapter sequences and specific primer sequences; C-primers: Common adapter primers contain common adapter sequences and specific primer sequences.

## Competing interests

Both authors declare that they have no competing interests.

## Authors’ contributions

DW developed the technique and drafted the manuscript. CZ supervised the project and revised the manuscript. All authors read and approved the final manuscript.

## Supplementary Material

Additional file 1 **Detection of genetic purity of a maize seed lot (200 seeds, Zhengdan 958).** The bands generated from the female parent of Zhengdan 958 are in red boxes, and the bands generated from the off-type seeds are in yellow boxes. Click here for file
