# Supplementary figures and images for: Universal Multiplex PCR: a novel method of simultaneous amplification of multiple DNA fragments
Source: Plant Methods. 2012 Aug 15;8:32. doi: 10.1186/1746-4811-8-32 (PMC3485162; doi:10.1186/1746-4811-8-32)

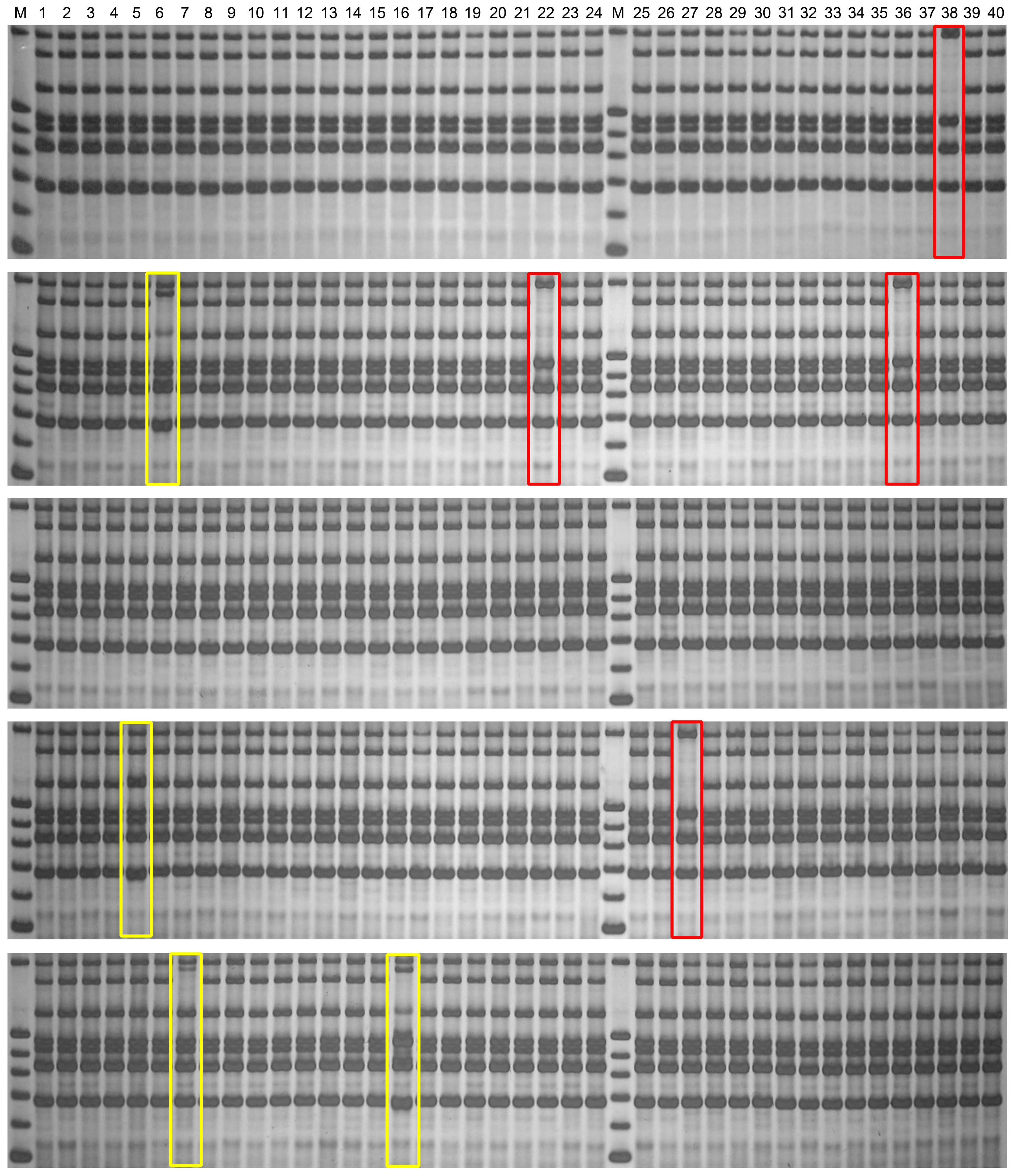

Supplement: Additional file 1 — Detection of genetic purity of a maize seed lot (200 seeds, Zhengdan 958). The bands generated from the female parent of Zhengdan 958 are in red boxes, and the bands generated from the off-type seeds are in yellow boxes. [file 1746-4811-8-32-S1.jpeg]
